# From Pilot Trap to Institutional Capacity: A Governance Framework for Sustainable Clinical AI Implementation in Health Systems

**DOI:** 10.2196/92680

**Published:** 2026-05-07

**Authors:** Jin Tian, Zengren Zhao, Longmei Tang, Yongzhao Song, Yuchang Li, Nan Jiang

**Affiliations:** 1 Hospital Management Innovation Center, The First Hospital of Hebei Medical University, 89 Donggang Street, Shijiazhuang, Hebei, 050000, China, +86 311 87156084; 2 Department of Social Medicine and Health Services Management, Hebei Medical University, Shijiazhuang, Hebei, China; 3 Hebei Province Key Laboratory of Environmental and Human Health, Shijiazhuang, Hebei, China; 4 Department of Public Health, The First Hospital of Hebei Medical University, Shijiazhuang, Hebei, China

**Keywords:** clinical artificial intelligence, AI governance, health systems, institutional infrastructure, implementation science, governance framework

## Abstract

Clinical artificial intelligence (AI) applications frequently fail to transition from short-term pilot projects into sustained components of routine clinical care, a phenomenon referred to in this viewpoint as the pilot trap. This persistent gap reflects not only technical or regulatory limitations but also insufficient governance capacity within health care organizations. We argue that such capacity is not fully established before deployment; rather, it develops through implementation as real-world operational tensions clarify organizational ownership, accountability boundaries, and coordination mechanisms. Drawing on an 18-month implementation of a provincial clinical AI platform in China, we develop a 6-module governance framework encompassing institutional carrier formation, infrastructure governance, regulatory and ethical governance, interdisciplinary coordination, translational scaling, and lifecycle evaluation and oversight. These modules represent functional governance conditions observed during implementation rather than a prescriptive institutional architecture to be installed prior to deployment. We further introduce the concept of functional transferability and position the framework as an upstream complement to existing international governance standards, which typically specify what governance should achieve, but often assume that the organizational capacity to implement it already exists. Advancing clinical AI beyond demonstration, therefore, depends less on model performance alone than on the ability of health systems to develop and sustain the institutional capacity required for routine clinical use.

## Introduction

Substantial investment in clinical artificial intelligence (AI) has generated a rapidly expanding portfolio of tools proposed for health care delivery, including applications for diagnostic support, clinical decision-making, and operational coordination [1,2]. Despite these advances, most clinical AI systems remain confined to short-lived pilots or proof-of-concept demonstrations rather than becoming durable components of routine care across health systems [3-5]. This persistent gap between technological progress and sustained clinical integration suggests that the central constraint lies not only in model performance but also in the institutional arrangements required to authorize, operate, and sustain AI as accountable clinical capacity. Understanding these institutional conditions therefore becomes central to explaining why technically promising AI systems fail to persist in routine clinical practice.

This pattern is frequently described as a “pilot trap,” in which technically promising systems fail to transition into stable operational use [4-7]. Under such conditions, ownership structures remain fragmented, accountability boundaries are unclear, and deployment depends on temporary resources or individual champions rather than durable institutional arrangements. What appears as a challenge of technological adoption therefore reflects a deeper problem of institutional capacity formation, not merely the absence of governance structures, but the absence of the implementation conditions through which such structures are generated and stabilized within routine clinical practice.

China provides an analytically informative setting for examining these dynamics. Clinical AI has been incorporated into national strategies for health care modernization and embedded within evolving regulatory frameworks governing software as a medical device [8,9]. Within this policy environment, provincial platforms and tertiary hospitals, where administrative legitimacy, clinical scale, and technical capacity converge, represent a structurally significant configuration for AI integration. These conditions make governance formation observable as a determinant of whether AI transitions from episodic deployment to institutional infrastructure. Yet, despite this policy alignment, the conditions enabling governance formation, including stable organizational ownership, standardized infrastructure, and sustained operational accountability, remain inconsistently realized.

Existing scholarship on clinical AI has largely concentrated on model validation, predictive performance, and application-level effectiveness [10-12]. Governance has increasingly been recognized as relevant to implementation outcomes, yet available frameworks remain limited in operational specificity [13-16]. High-level governance principles often lack clearly defined organizational carriers through which governance becomes actionable [17], while implementation-oriented approaches tend to emphasize evaluation and oversight without addressing upstream conditions of ownership, financing, and sustained operational responsibility [6,18]. Critical questions therefore remain underspecified, including who governs clinical AI, under what institutional mandate, and how accountability is distributed across the system lifecycle. Without addressing these questions, the institutional conditions required for sustained clinical AI integration remain poorly understood.

In this viewpoint, we draw on an 18-month real-world implementation of a provincial clinical AI platform in China to develop a governance framework for the institutionalization of clinical AI. By analyzing contemporaneous implementation materials, including governance documents, regulatory filings, and operational records, we identify 6 interdependent governance modules that repeatedly emerged as necessary conditions for sustained deployment. These modules describe the organizational and governance capacities required for clinical AI to transition from episodic pilot projects to routinized clinical infrastructure. We further introduce the concept of functional transferability to distinguish governance functions that remain stable across contexts from the institutional forms through which they may be realized. The framework is intended both to explain why many clinical AI systems remain pilot-bound and to help identify governance conditions that support durable AI integration across health systems.

## Conceptual Framing

The conceptual framing of this study is grounded in 3 interrelated constructs: institutionalization, infrastructure, and governance. Institutionalization refers to the stabilization of AI-enabled practices as routinized clinical processes that persist beyond pilot deployments and project-based implementation. Infrastructure denotes the technical and operational substrate required for reliable and repeatable system use, encompassing data pipelines, interoperability systems, computational capacity, and access control. Governance refers to the allocation of decision rights and accountability arrangements that authorize system use, assign responsibility, and oversee system updates across the deployment lifecycle [19,20].

These constructs are analytically distinct yet operationally sequential. Infrastructure constitutes the technical substrate that enables system operation. Governance organizes the human and institutional arrangements through which that substrate is authorized, monitored, and maintained. Institutionalization denotes the observable outcome that emerges when governed systems persist in routine use over time. Governance and institutionalization are related but not identical. Governance refers to the arrangements through which accountability and oversight are structured, whereas institutionalization denotes the condition that becomes observable when such arrangements stabilize sufficiently to sustain routine practice beyond project-specific resources or individual champions. Governance may exist without institutionalization having yet been achieved, whereas institutionalization presupposes the presence of governance arrangements capable of sustaining routine practice.

**Figure 1.**
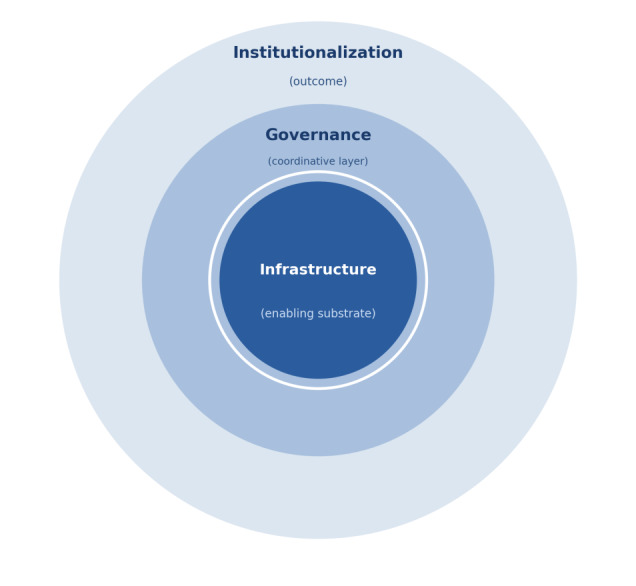
Infrastructure provides the technical substrate that enables system operation, including data, computing, and interoperability capacities. Governance organizes coordination and accountability across the lifecycle of deployment by structuring authorization, oversight, and operational responsibility. Institutionalization represents the sustained integration of artificial intelligence–enabled practices into routine clinical workflows as governed systems persist over time. The figure illustrates the sequential relationship through which infrastructure enables governance and governance supports institutionalization.

In this study, these constructs are not treated as fully established preconditions prior to implementation. Governance capacity in particular is conceptualized as emergent rather than antecedent. It develops through the operational demands generated during implementation rather than being fully specified in advance. Implementation processes therefore play a constitutive role in shaping governance arrangements, through which accountability structures, decision rights, and operational responsibilities gradually become stabilized within clinical environments [21]. Institutionalization emerges when these governance arrangements persist and sustain routine clinical practice over time. The sequential relationship among these 3 constructs is illustrated in Figure 1.

## Case Context

To examine how the conceptual relationships outlined above operate in practice, this study analyzes the implementation of a clinical AI platform at a large tertiary academic hospital in Hebei Province, China. Over an 18-month period, the institution deployed a set of AI-enabled systems aimed at supporting both clinical decision-making and operational coordination across departments and affiliated institutions. Implementation was organized around 3 clinical pathways, encompassing intelligent preconsultation triage, oncology multidisciplinary team (MDT) decision support, and therapeutic drug monitoring (TDM). Each pathway represents a domain in which AI systems intersect directly with routine clinical workflows and institutional decision structures and therefore provides an observable setting for examining governance formation during deployment.

The hospital functions as a regional medical center and maintains substantial digital infrastructure, including integrated clinical information systems and data management capabilities. As a large tertiary hospital operating within China’s national regulatory framework for AI-enabled medical technologies, it represents a setting where clinical scale, digital infrastructure, and regulatory oversight converge. This institutional configuration creates an analytically valuable context for observing how governance arrangements evolve as AI systems transition from experimental deployment to sustained integration within routine clinical practice.

## From Implementation Materials to Governance Framework: Analytical Approach

This study adopts a qualitative, case-based process-tracing approach to examine how governance structures emerged during an 18-month clinical AI platform implementation at a large tertiary academic hospital in Hebei Province, China. The analytical strategy follows qualitative case study logic commonly used to examine institutional processes, organizational coordination, and decision trajectories within complex health system settings.

Empirical materials were derived from institutional artifacts generated through routine platform development and deployment. These materials comprised 17 institutional artifacts, including 13 text-based documents and 4 visual implementation materials, encompassing governance frameworks, regulatory filings, infrastructure certification records, internal operational monitoring records, operational deployment artifacts, monthly work reports, meeting materials, organizational documents, budget requests, workflow diagrams, and platform architecture and feature maps. These materials documented platform development milestones, coordination events, governance decisions, and operational activity across the review period from July 2, 2024, to January 24, 2026. Internal operational monitoring records additionally provided aggregated operational indicators documenting system use during platform deployment. These indicators were used descriptively to characterize continuity of deployment, scope of workflow embedding, and organizational consolidation rather than as formal effectiveness endpoints.

Materials were organized chronologically to identify key decision points and coordination tensions, defined as documented conflicts, delays, or breakdowns requiring institutional responses. Governance modules were derived inductively from recurrent coordination tensions and the institutional mechanisms developed to resolve them. Data analysis followed an inductive thematic analysis approach in which coordination tensions identified across implementation artifacts were iteratively grouped into higher-level governance functions that subsequently informed the 6-module framework. A tension was considered governance-relevant when it recurred across multiple implementation episodes and was corroborated by more than one category of institutional artifact. Initial coding and module development were conducted by two authors and subsequently reviewed by team members with distinct clinical, administrative, and policy perspectives to strengthen interpretive robustness and reduce the risk of post hoc rationalization. Members of the research team participated in documenting institutional processes during the implementation period, enabling access to contemporaneous institutional materials while maintaining analytical separation from AI model development, technical validation, and system performance evaluation activities. Coding was conducted manually by the authors without the use of qualitative data analysis software.

This retrospective, case-based study received formal approval from the Clinical Research Ethics Committee of The First Hospital of Hebei Medical University (approval number: 2026; research review number: 032; approval date: February 5, 2026). The analysis reported in this manuscript was based on institutional documents and aggregated operational materials and did not involve the use of identifiable individual patient-level data.

## Governance Framework for Clinical AI Institutionalization

### Development of the Governance Framework

Analysis of implementation materials, conducted through the process-tracing approach described above, identified recurrent operational tensions arising during the deployment of clinical AI systems. These tensions were primarily associated with infrastructure integration, regulatory alignment, and the embedding of AI outputs within routine clinical workflows. Recurrent solutions to these implementation tensions were grouped into 6 interdependent modules. These modules represent governance conditions observed in practice rather than a prescriptive institutional design.

**Figure 2.**
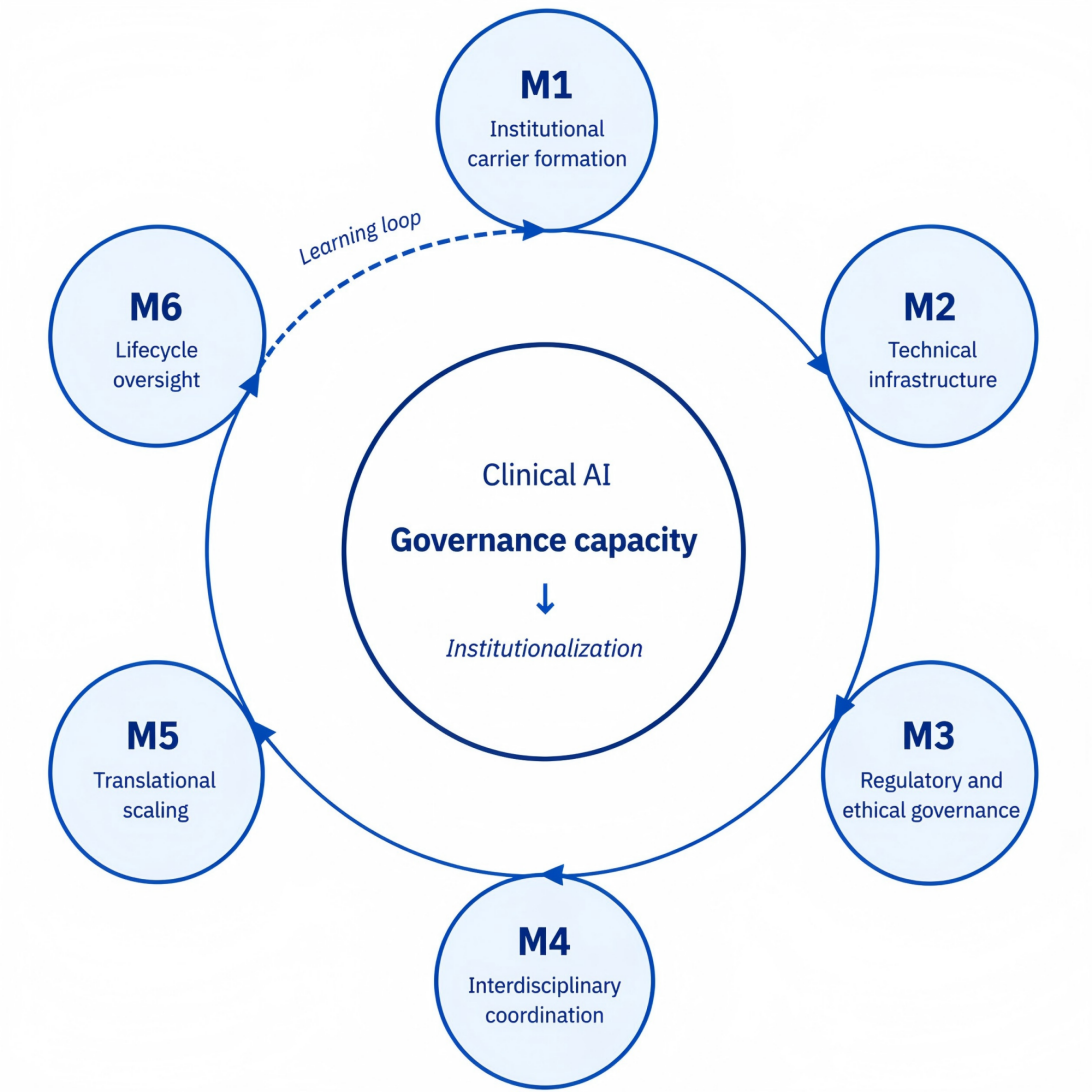
Six governance modules (M1-M6) represent organizational, technical, regulatory, and clinical functions required for sustained AI deployment. These modules collectively constitute governance capacity that enables the transition of clinical AI systems from pilot initiatives to institutionalized clinical practice. The framework operates as an adaptive learning cycle in which lifecycle oversight informs continuous governance formation. AI: artificial intelligence.

The 6 modules collectively constitute a governance cycle through which clinical AI systems transition from experimental deployment to stabilized institutional use. The first two modules, institutional carrier formation and infrastructure governance, represent upstream enabling conditions that establish organizational ownership and sustain technical continuity prior to clinical activation. Regulatory and ethical governance defines authorization boundaries and distributes accountability across the deployment lifecycle. The remaining modules, interdisciplinary operational coordination, translational scaling, and lifecycle evaluation and oversight, support active deployment by enabling cross-departmental embedding, contextual expansion, and adaptive governance as clinical and regulatory conditions evolve. Although analytically presented as 6 modules, the framework operates as a recursive governance cycle in which implementation generates feedback that informs subsequent governance adjustments and institutional learning. The governance framework is illustrated in Figure 2.

### Overview of the Governance Framework

The framework comprises 6 interdependent modules representing the organizational, technical, regulatory, and clinical functions required to stabilize AI systems as routinized components of clinical practice. Each module reflects a recurring governance tension identified during implementation and is supported by empirical process evidence drawn from the Hebei provincial clinical AI platform. Table 1 summarizes the modules, their corresponding implementation artifacts, and the transferable governance requirements derived from them.

**Table 1.** Governance framework modules, empirical implementation artifacts, and transferable governance requirements.

Governance module	Empirical evidence from implementation	Transferable governance requirement
M1: Institutional carrier formation	Establishment of a dedicated medical AI^a^ laboratory through formal administrative orders; defined organizational structure integrating algorithm development, clinical research, and application translation; designation as a provincial engineering research center.	Clinical AI deployment requires a designated institutional carrier with formal mandate, decision authority, and organizational independence from temporary project teams.
M2: Infrastructure governance	Integration of high-performance computing infrastructure with hospital information systems; EMR^b^ functional maturity certification; interoperability standardization assessments; documented institutional data governance policies.	Data pipelines, computational capacity, and interoperability mechanisms must be governed as durable institutional infrastructure with defined access, security, and update controls.
M3: Regulatory and ethical governance	Algorithm filing under the national regulatory framework administered by the Cyberspace Administration of China; documentation defining the system’s purpose, use boundaries, and institutional accountability.	Regulatory authorization and accountability structures should be formally specified prior to clinical activation of AI systems.
M4: Interdisciplinary operational coordination	Three-division laboratory governance structure; integration of AI outputs into multidisciplinary tumor board workflows; documented role allocation and decision rights across clinical and technical teams; temporary suspension of a pathway pending clarification of update authority.	Operational deployment requires clearly defined roles, decision rights, and accountability structures linking algorithm development, clinical decision processes, and institutional oversight.
M5: Translational scaling	Expansion of the AI-supported preconsultation pathway across multiple outpatient departments; cross-department workflow integration protocols; establishment of a provincial clinical AI research institute supporting broader deployment.	Scaling requires governance pathways that translate localized operational success into institution-wide organizational capability.
M6: Lifecycle evaluation and oversight	Documentation of system use boundaries; role-based pathway supervision; model version update logs; institutional review of operational feedback during implementation.	Lifecycle governance requires monitoring mechanisms tied to institutional authority to manage system updates, operational feedback, and evolving clinical conditions.

a AI: artificial intelligence.

b EMR: electronic medical record.

Across the observation period, the platform was progressively integrated into routine clinical workflows spanning multiple service lines. Risk assessment tools were incorporated into outpatient triage processes to support early identification of high-risk patients, while decision-support functions were embedded within multidisciplinary case discussions to facilitate structured cross-departmental evaluation. AI-assisted documentation tools, including generative medical record support and natural-language clinical assistants, were concurrently introduced to support routine documentation and information retrieval. The platform also enabled structured capture of research indicators and integration of clinical data management with institutional quality-monitoring functions. To provide high-level proof-of-concept evidence that the platform progressed beyond bounded pilot deployment, selected operational indicators are summarized in Table 2.

**Table 2.** Reported operational indicators and measurement definitions^a^.

Indicator	Definition and measurement basis	Observed value
Cumulative patient interactions (preconsultation pathway)	Total number of completed patient-initiated interactions recorded in system logs across all 5 enrolled outpatient departments from the first departmental onboarding to the end of the observation period	>24,000
Recent average daily use	Mean number of completed interactions per calendar day calculated over the postrollout operational phase, following phased onboarding of all 5 departments	~110 encounters/day
Continuous operational duration (preconsultation pathway)	Number of consecutive months the pathway remained operationally active following departmental rollout	>12 months
Number of clinical pathways in sustained deployment	Count of artificial intelligence–enabled clinical pathways that remained in active institutional use across the focal observation period	3

a The operational indicators reported in this table were derived from aggregated institutional operational monitoring records generated during routine platform deployment. They are presented to improve interpretive transparency rather than as indicators of clinical effectiveness. The recent daily use figure refers to the mature postrollout phase following phased departmental onboarding and should not be interpreted as a rate that applied uniformly across the entire cumulative review period. No identifiable individual patient-level data were used.

### Empirical Context of Platform Operation

During the observation period, the clinical AI platform was progressively integrated into routine clinical workflows across multiple service lines. Operational deployment was organized around 3 clinical pathways, each representing a domain in which AI-assisted functions were embedded within routine clinical decision processes and institutional coordination mechanisms.

The intelligent preconsultation pathway deployed a conversational interface based on a hospital-developed and locally deployed large language model–enabled system, rather than a commercial vendor platform, to elicit structured symptom histories from patients prior to clinical encounters. The pathway generated summarized risk assessments that were automatically transmitted to the electronic medical record (EMR) system, thereby providing preliminary clinical information to support outpatient triage and physician assessment at the point of care.

The oncology multidisciplinary decision-support pathway embedded AI-assisted treatment recommendations within tumor board workflows. It was supported by a hospital-developed oncology decision-support model based on a transformer architecture and adapted for oncology-specific text generation and clinical recommendation tasks. Model-generated outputs were reviewed alongside imaging findings, pathology reports, and patient-specific clinical data under defined specialist decision authority, with final treatment determinations remaining within established clinical accountability structures.

The TDM pathway applied an in-house machine learning pipeline that integrated principal component analysis for feature optimization and a transformer-based deep learning regression model to generate plasma drug concentration estimates for duloxetine and support individualized dosing decisions. These estimates were derived from patient-specific clinical parameters and incorporated into routine medication-management workflows to inform dosing adjustments.

In addition to these clinical pathways, AI-assisted documentation tools were deployed to support routine information management, including generative medical record functions and natural-language clinical assistants facilitating clinical documentation and information retrieval. The platform also enabled structured capture of research indicators and integration of clinical data management with institutional quality-monitoring and downstream analytical processes. Sustained operation across these domains created an empirical context in which AI-enabled systems engaged directly with institutional governance arrangements and cross-departmental coordination structures. This provided the empirical basis for examining how governance mechanisms emerged and evolved during real-world implementation.

Three forms of implementation evidence were observable during the review period, together indicating that the platform had moved beyond bounded pilot deployment. Sustained operation was evident across 3 clinical pathways, with the preconsultation pathway remaining in routine use for more than 12 months and recording more than 24,000 completed patient interactions across 5 outpatient departments. At the same time, AI outputs were incorporated into existing clinical workflows rather than remaining external demonstrations, including automatic transmission of preconsultation summaries to the EMR system, integration of oncology decision support into MDT deliberation, and incorporation of duloxetine concentration prediction into medication-management processes. Continuity of deployment was also accompanied by formal governance consolidation: regulatory authorization was documented in the algorithm filing record, role-based operational oversight was specified in pathway supervision protocols, update procedures were recorded in version-control logs, and organizational ownership was formalized through the medical AI laboratory mandate. These observations collectively provide high-level implementation evidence that the platform functioned as an institutionalized operational capability rather than remaining a time-limited pilot initiative.

### Institutional Carrier Formation

A foundational governance challenge in clinical AI deployment concerns the absence of a stable organizational locus of ownership. Under such conditions, the institutional authority required to coordinate development, deployment, and lifecycle oversight remains fragmented, preventing AI capabilities from transitioning beyond pilot implementation into durable institutional use.

When AI initiatives operate as project-based activities, accountability for cross-departmental data access, clinical validation, and regulatory engagement becomes diffusely distributed across organizational units and external collaborators. In the absence of a designated institutional carrier, technically functional systems frequently fail to persist beyond initial pilot phases because no organizational entity possesses the authority required to align technical development with clinical governance and institutional decision structures.

Governance consolidation in the studied setting occurred through the establishment of a dedicated medical AI laboratory serving as an institutional carrier with a formal mandate and defined decision rights. The laboratory integrated algorithm development, clinical research, and application translation within a unified organizational structure, thereby concentrating responsibility for AI system oversight within a single accountable entity. This carrier was established prior to broader clinical integration and subsequently provided the administrative foundation upon which additional governance arrangements were constructed, including designation as a provincial engineering research center.

Institutional arrangements may vary across health systems in organizational form and administrative configuration. A consistent governance principle nevertheless emerges, whereby sustained clinical AI integration requires a durably designated organizational entity responsible for coordinating development, deployment, and lifecycle oversight. Such carriers may take the form of dedicated laboratories, formally chartered governance committees, or federated institutional arrangements, but must remain structurally distinct from temporary project teams and independent of short-term funding cycles.

### Infrastructure Governance

A second governance challenge arises from the treatment of data and computational resources as project-bound technical assets rather than as governed institutional infrastructure. When infrastructure provision remains embedded within temporary project arrangements, the standardization, interoperability, and operational continuity required for routine clinical deployment cannot be reliably sustained. Under such conditions, AI systems remain dependent on configurations that are structurally incompatible with long-term institutional operation.

Fragmented information systems, inconsistent data standards, and reliance on temporary computing environments constrain stable model operation and impede integration into clinical workflows. These limitations typically arise not from data scarcity but from the absence of institutional governance mechanisms capable of ensuring infrastructure continuity, access control, and system-wide coordination across heterogeneous hospital environments. Without formal governance of underlying infrastructure, technically validated AI systems may nevertheless fail to operate reliably at the point of clinical deployment, creating conditions in which deployment continuity depends on informal negotiation rather than defined accountability structures.

Infrastructure governance within the studied platform was progressively incorporated into institutional oversight arrangements. High-performance computing capacity was established and integrated with existing clinical information systems, while external certifications, including EMR functional maturity assessment and interoperability standardization evaluation, confirmed that infrastructure conditions satisfied requirements for clinical deployment. Data standardization across legacy systems further required iterative coordination among clinical, technical, and administrative units, as nonstandardized terminologies embedded within departmental workflows impeded the establishment of unified data pipelines.

Infrastructure architectures differ across institutions in scale, technical configuration, and organizational management. A consistent governance principle nevertheless emerges, whereby data pipelines, computational resources, and interoperability systems must be governed as durable institutional infrastructure, maintained under defined accountability arrangements and independent of the project-bound funding cycles that characterize early-stage AI deployment.

### Regulatory and Ethical Governance

A further governance challenge concerns the specification of regulatory authorization and accountability prior to clinical deployment. When regulatory alignment is deferred until after deployment decisions are made, accountability arrangements remain incompletely defined during the period of greatest institutional uncertainty, and the boundaries of legitimate system use are established retrospectively rather than by design.

In the absence of prior regulatory specification, system purpose, intended use boundaries, and designated institutional responsibility may remain insufficiently defined at the point of clinical activation. Effective governance therefore requires that regulatory authorization and accountability structures be established prior to deployment, particularly in regulatory environments that continue to evolve alongside emerging clinical AI capabilities.

Within the Hebei provincial platform, regulatory authorization was obtained prior to broader clinical activation of the oncology decision-support pathway through national algorithm filing procedures administered under the Cyberspace Administration of China framework. The filing process clarified system purpose, defined intended use boundaries, and formally designated institutional responsibility for ongoing deployment. Regulatory requirements therefore informed operational design decisions at the predeployment stage rather than constraining systems already embedded in clinical practice, and oversight functions were incorporated within existing institutional governance arrangements rather than constituted as a separate compliance structure.

Regulatory frameworks vary substantially across jurisdictions in scope, procedural requirements, and institutional applicability. A consistent governance principle nevertheless emerges, whereby accountability specification and the definition of authorized use boundaries should precede clinical activation. When regulatory alignment occurs only after deployment, governance uncertainty persists during implementation and institutional oversight across the system lifecycle becomes structurally constrained.

### Interdisciplinary Operational Coordination

Effective clinical deployment of AI systems depends on structured coordination across clinical, technical, and administrative domains. When such coordination is not institutionally specified, technically functional systems may operate without agreed procedures for clinical use, and accountability for AI-generated outputs may remain diffuse or unassigned.

Ambiguity frequently emerges at the interface between algorithm development and clinical decision-making. Technical personnel may not possess the authority to modify systems in response to operational feedback, while clinicians may lack formally defined procedures for incorporating AI outputs into diagnostic or therapeutic workflows. Without governance arrangements that connect these domains, AI systems may remain technically operational but institutionally unstable.

Operational coordination in the studied implementation was institutionalized through explicit role allocation across a 3-division laboratory structure responsible for algorithm development, clinical research, and application translation. In the oncology multidisciplinary decision-support pathway, AI outputs were embedded within established team workflows under defined decision rights. Model-generated outputs were reviewed during structured multidisciplinary meetings alongside imaging findings, pathology reports, and patient-specific clinical data, while final treatment decisions remained within established clinical accountability structures. Early deployment further revealed that responsibility boundaries required clarification, and one pathway experienced temporary suspension pending resolution of ambiguity regarding authority over model update decisions.

Institutional coordination mechanisms differ across health systems in organizational design and governance structure. A consistent governance principle nevertheless emerges, whereby sustained clinical deployment requires clearly defined roles, decision rights, and accountability structures linking algorithm development, clinical use, and institutional oversight.

### Translational Scaling

Durable institutional integration of clinical AI systems requires governance mechanisms capable of extending operational capacity beyond localized implementations. Without such mechanisms, systems demonstrating reliable performance within circumscribed clinical contexts may remain confined to isolated deployments, preventing broader organizational adoption and durable integration at scale.

Localized operational success does not automatically generate the institutional authority required for expansion across departments or clinical service lines. Effective scaling therefore depends on governance arrangements that connect deployment experience with institutional decision structures, enabling operational practices developed in one context to be translated into formally recognized organizational capabilities.

In the studied implementation, scaling occurred through progressive integration of AI-supported pathways into institutional governance structures rather than replication of isolated applications. The preconsultation risk assessment pathway was extended across multiple outpatient departments through standardized workflow integration, enabling its routine use across distinct clinical service lines. Additional AI-supported pathways were subsequently introduced in coordination with clinical and administrative units, each accompanied by governance documentation specifying operational roles, workflow procedures, and system use parameters. Over time, these expansions transformed initially localized tools into institutionally coordinated clinical services embedded within routine care processes. Institutional recognition further reinforced this process through research center designation and the establishment of a provincial clinical AI research institute.

Scaling mechanisms vary across institutional contexts in governance structure and policy environment. A consistent governance principle nevertheless emerges, whereby durable expansion of clinical AI capacity requires governance pathways linking localized deployment with institution-wide authority structures, allowing operational experience to translate into formally recognized institutional capability.

### Lifecycle Evaluation and Oversight

Long-term clinical deployment of AI systems requires governance mechanisms capable of monitoring performance, managing system updates, and adapting deployment conditions over time. Without such mechanisms, operational systems may gradually diverge from their intended use conditions as clinical workflows change, data distributions shift, and regulatory expectations develop over time.

In the absence of structured lifecycle oversight, responsibility for monitoring system performance may become diffuse across organizational units. Version control may be inconsistently documented, and operational feedback generated by clinical users may not systematically reach those with authority to modify deployed systems. This configuration introduces the risk of operational drift as AI systems expand beyond initial pilot environments into routine clinical practice, underscoring the necessity of governance mechanisms capable of maintaining accountability across the full system lifecycle.

Within the observed implementation, lifecycle oversight practices were incorporated into existing institutional governance arrangements rather than established as a fully separate governance protocol. Monitoring activities included documentation of system use boundaries, clarification of responsible units for pathway supervision, and iterative adjustments in response to operational feedback. System updates were conducted within established institutional decision structures and aligned with regulatory requirements. Standardized version-control documentation remained under iterative development during the observation period as institutional governance procedures matured.

Lifecycle governance arrangements vary across institutional contexts in their degree of formalization and organizational structure. Across these variations, a consistent governance principle applies, whereby sustained clinical deployment requires monitoring mechanisms linked to defined institutional authority, ensuring that system updates, clinical workflow adjustments, and evolving regulatory expectations remain subject to accountable oversight throughout the operational lifecycle.

## Discussion

### From Implementation to Governance Formation

Persistent pilot dependence in clinical AI is commonly attributed to governance deficits, including the absence of accountability structures, ownership arrangements, or regulatory alignment that would enable AI systems to move from experimental deployment into routine clinical use [22,23]. While this interpretation identifies important institutional constraints, it does not fully explain why governance arrangements so often fail to emerge even when pilot implementations are technically successful. The present analysis points to a more precise formulation. In this view, the pilot trap reflects not only the absence of governance but also insufficient implementation depth for governance structures to take shape [4,24]. Systems remain project-bound not because governance was unavailable in principle, but because deployment ended before the operational tensions capable of generating governance had fully materialized. What distinguishes sustained integration from persistent pilot dependence is therefore not the prior existence of a governance framework but the capacity of implementation processes to generate governance through iterative responses to recurrent coordination demands.

Reconceptualizing the pilot trap in institutional terms carries direct implications for how it is addressed. Existing approaches often position governance as a precondition for deployment by emphasizing accountability frameworks, compliance structures, and ownership arrangements before clinical integration proceeds [25,26]. Evidence from this case indicates a different dynamic. Governance capacity did not precede implementation; it emerged through it. Data-standardization conflicts, responsibility-boundary disputes, and scaling misalignments created the institutional conditions under which governance structures became necessary and were progressively consolidated [27]. Deployment therefore proceeded not from a complete governance architecture but from a minimal institutional foundation that accumulated governance capacity as implementation deepened. In contrast to frameworks that emphasize technical integration requirements or clinician adoption barriers as the primary constraints on clinical AI sustainability, the present analysis foregrounds the governance formation process itself. Attention is directed toward the mechanisms through which organizational ownership, accountability boundaries, and coordination structures become stabilized as durable institutional capacity. Across the implementation trajectory, 6 governance modules repeatedly emerged as functional conditions necessary for sustained deployment rather than as elements of a predefined governance design. Considered together, these modules constitute governance capacity as a system-level capability through which sociotechnical resources are consolidated into routinized clinical infrastructure. The operational indicators summarized in Table 2 provide empirical grounding for this interpretation and suggest that governance formation was accompanied by sustained operational embedding rather than remaining a purely organizational or aspirational construct.

These indicators are not reported as performance benchmarks but as governance-relevant evidence. Sustained use across more than 12 months of continuous operation, encompassing more than 24,000 patient interactions within a phased multidepartment deployment, indicates that governance arrangements were sufficient to support accountable clinical operation over time rather than confining the platform to a bounded pilot episode. Operational continuity across the preconsultation pathway is consistent with the stabilization of M1 and M2, without which system continuity would be more likely to depend on informal coordination than on defined accountability structures. Regulatory authorization obtained prior to clinical activation of the oncology decision-support pathway is consistent with the functioning of M3, as documented in the algorithm filing record and related compliance materials. The maintenance of defined clinical decision authority throughout the oncology MDT pathway is consistent with the operationalization of M4. The phased expansion of the preconsultation pathway across multiple outpatient departments is indicative of M5, as documented in deployment materials and departmental rollout records. Iterative system updates conducted within institutional governance structures during the observation period are indicative of M6 functioning as an active governance mechanism rather than a nominal policy commitment. Although governance functions vary in their observability through use metrics, each of the 6 modules is linked to at least one category of traceable institutional artifact, including operational records, regulatory filings, deployment materials, and organizational documents. This supports the descriptive reproducibility of the framework as an analytical lens, whereas its broader transferability across heterogeneous health system contexts is discussed in the following section. Collectively, these indicators suggest that the governance modules described in this framework were associated with observable operational continuity and that the transition from pilot deployment to institutionalized clinical practice was sustained across multiple service lines and governance domains.

### From Implementation Tensions to Governance Mechanisms

Implementation of the platform was accompanied by several operational frictions that required institutional responses, through which governance structures gradually emerged [24,28]. Rather than resulting from deliberate institutional design, governance arrangements developed through iterative responses to practical operational challenges. The analysis identifies 3 generative mechanisms that shaped this process: authorization, responsibility allocation, and coordination.

Authorization translated regulatory expectations into operationally defined deployment boundaries and clarified the legitimate scope and conditions under which clinical AI systems could be used [25,29]. Responsibility allocation embedded AI-related tasks within existing organizational structures by assigning accountable ownership across institutional units and professional roles [30]. Coordination across clinical, technical, and administrative domains was gradually stabilized as decision rights, communication channels, and operational interfaces became more clearly defined [31].

These mechanisms emerged through concrete operational friction rather than prior institutional design. During early deployment of the TDM pathway, inconsistencies in laboratory data formatting and record structures across hospital information systems temporarily limited automated data ingestion and required manual reconciliation. Resolving this tension required sustained coordination among clinical departments, the hospital information center, and the AI development team, ultimately producing standardized data interfaces and validation procedures, which were later incorporated into the governance framework.

A second coordination tension arose during deployment of the oncology MDT decision-support pathway. Clinician uncertainty regarding the appropriate role of AI-generated recommendations within established multidisciplinary decision-making processes led to a temporary operational suspension. Following administrative review, responsibility boundaries and documentation requirements were clarified, and the governance framework formalized AI outputs as advisory inputs under defined clinician oversight with role-based monitoring arrangements.

These episodes illustrate that institutionalization does not occur through seamless technological integration but through the iterative resolution of organizational tensions [27,32]. Governance neither simply precedes implementation nor follows it as a downstream regulatory layer. Instead, governance is produced through the implementation process itself. Institutionalization emerges when governance arrangements stabilize in response to recurrent operational demands, transforming iterative problem-solving into durable institutional capacity embedded within routine organizational practice.

### From Governance First to Governance Fit

Effective clinical AI deployment requires governance fit rather than governance expansion. Governance-first approaches are sometimes criticized for introducing procedural rigidity that slows technological innovation. This analysis addresses that concern by distinguishing governance functions from the institutional forms through which they are implemented. This distinction also clarifies why the framework is analytically reproducible across settings even when organizational forms differ.

Certain governance functions constitute nonnegotiable conditions for safe and accountable integration. These include clearly defined authorization boundaries, accountable ownership, and mechanisms for lifecycle oversight. The institutional arrangements through which such functions are enacted, however, may vary substantially in scale, procedural intensity, and degree of centralization across health system contexts. Accordingly, reproducibility in this context lies not in replicating identical organizational structures but in assessing whether comparable governance functions can be identified through traceable institutional evidence.

Governance fit refers to the calibrated alignment between governance intensity and system characteristics such as clinical risk, update frequency, and operational complexity [25,33]. Under this perspective, governance architectures should not impose uniform procedural requirements across all deployment environments. Instead, effective governance preserves adaptive capacity by calibrating oversight intensity according to the potential consequences and reversibility of system changes.

Several mechanisms illustrate how such calibration can be operationalized in practice. Risk-tiered update thresholds allow routine model refinements to proceed under simplified authorization procedures while reserving stricter review processes for high-impact system modifications. Predefined authorization pathways for low-risk adjustments further reduce procedural bottlenecks. Staged deployment strategies similarly enable systems to evolve within monitored operational environments before wider clinical integration [34].

Both extremes generate systemic challenges. Insufficient governance creates fragmentation and reinforces persistent pilot dependence, whereas excessive governance may introduce bureaucratic inertia that constrains technological adaptation [35,36]. Health systems can therefore balance accountability with technological agility by implementing governance arrangements that differentiate routine system evolution from high-risk modification processes while maintaining clear institutional responsibility for oversight. The broader question of how these governance functions travel across institutional environments is addressed in the following section on functional transferability.

### From Clinician-Centric to Participatory Governance Maturation

The absence of structural patient and community involvement in the present case invites a more precise analytical observation than simply noting a gap. Existing literature on patient participation in clinical AI often conflates two distinct roles. Patients may participate as design informants who help shape decisions about system purpose, or as governance stakeholders who contribute to oversight of how deployed systems operate within clinical environments [37,38]. Most existing guidance addresses the former by emphasizing co-design principles and participatory development methodologies. By contrast, patient involvement in operational governance has received substantially less analytical attention, and the institutional conditions required to make such participation structurally meaningful remain insufficiently specified.

Participatory governance capacity is not a function of inclusion per se but of the institutional anchoring through which participatory inputs acquire an accountable pathway to governance decisions. Meaningful patient involvement in AI governance requires an organizational carrier capable of receiving, processing, and acting on participatory inputs within established responsibility structures. Without such a carrier, participation risks becoming consultative rather than consequential, producing inputs that lack a defined institutional pathway to governance decisions [39]. The sequencing observed here, therefore, reflects a structural constraint rather than a deliberate exclusion. Participatory mechanisms were introduced following initial stabilization of the accountability architecture, a sequencing that reflected the specific institutional conditions of this implementation context rather than a principled position on the optimal timing of patient involvement. Contemporary co-design frameworks and implementation science literature frequently advocate for patient involvement from the earliest stages of governance development, and parallel co-design of governance and participation structures represents a well-documented and viable alternative in other health system contexts.

As institutionalization advances and governance architecture stabilizes, participatory capacity becomes both feasible and functionally necessary. Within the proposed framework, patient and community involvement is most appropriately integrated into lifecycle governance mechanisms. Defined feedback channels, transparent communication of system use boundaries, and structured review of operational incidents allow oversight to extend beyond professional actors while preserving clear lines of institutional accountability [38,40]. This integration addresses not only the legitimacy requirements identified in the literature but also the operational question of how participatory inputs are processed within functioning governance structures. Design-focused participation frameworks rarely specify these institutional pathways, leaving the governance dimension of participation largely unresolved [37,41].

### From Structural Replication to Functional Transferability

Although the framework emphasizes governance capacity rather than technological sophistication, several baseline conditions support its applicability across health system contexts [42]. These conditions represent minimal enabling environments for sustained clinical AI operation rather than structural requirements of the framework itself. Institutional authority to assign responsibility for AI oversight must be present. Basic digital infrastructure capable of supporting clinical data capture, storage, and computational processing is also required, together with regulatory or administrative mechanisms that permit AI tools to be activated within clinical workflows. Interdisciplinary coordination capacity must additionally connect clinical practice, technical development, and organizational management to sustain cross-functional collaboration. Where such conditions are only partially present, functional equivalents such as shared infrastructure platforms, external technical partnerships, or regional governance arrangements may provide alternative implementation pathways [43].

Framework transferability therefore lies in functional reproduction rather than structural replication [22]. In this paper, functional transferability is used as an analytic formulation to distinguish transferable governance functions from the setting-specific organizational forms through which they are instantiated. Institutionalization does not require identical organizational architectures but depends on the presence of governance functions capable of stabilizing clinical AI as durable institutional capacity. Six functional requirements underpin this process, including durable ownership arrangements, governed infrastructure, defined authorization boundaries, cross-functional coordination mechanisms, structured scaling pathways, and lifecycle oversight. Under this interpretation, governance transferability depends on whether equivalent institutional functions can be realized through different organizational arrangements across settings, rather than on replication of a single structural form. In this sense, reproducibility should be understood descriptively rather than structurally. What can be reproduced across settings is not an identical organizational form but an analytic approach that examines whether comparable governance functions are evidenced through traceable institutional artifacts. Table 3 illustrates how these requirements may be instantiated across heterogeneous health system contexts. It presents both the institutional arrangements observed in the study setting and illustrative analogs that may fulfill equivalent governance roles in more decentralized health systems. The purpose of this mapping is therefore to operationalize functional equivalence across contexts, rather than to prescribe identical organizational structures.

**Table 3.** Functional governance requirements and cross-system analogs.

Governance module	Functional requirement	Example instantiation in study context	Possible analog in decentralized health systems
M1: Institutional carrier formation	Accountable ownership formally assigned beyond project teams with defined decision authority across deployment, maintenance, and update cycles.	Provincial engineering research center and hospital AI^a^ laboratory.	Health system AI governance office or clinical AI steering committee.
M2: Infrastructure governance	Data and computational resources governed as institutional infrastructure ensuring continuity of access and interoperability with clinical systems.	Institutionally governed data platforms and high-performance computing environment.	Cloud-based infrastructure operating under institutional data governance agreements.
M3: Regulatory and ethical governance	Predeployment authorization establishing legal legitimacy and defined accountability boundaries for clinical AI use.	National algorithm filing and institutional compliance documentation.	Software-as-a-medical-device approval pathways and institutional compliance review processes.
M4: Interdisciplinary coordination	Stable cross-functional coordination with defined roles and decision rights linking clinical, technical, and administrative actors.	Three-division laboratory integrating clinical, technical, and translational roles.	Cross-functional clinical AI committees linking clinicians, engineers, and administrators.
M5: Translational scaling	Governance mechanisms linking localized deployment with higher-level institutional authority to support controlled expansion.	Regional medical alliances extending AI services across affiliated hospitals.	Multisite health system networks or collaborative implementation consortia.
M6: Lifecycle evaluation and oversight	Sustained monitoring and controlled system evolution through performance review, version control, and governance adjustment mechanisms.	Role-based monitoring and documented change-control procedures.	Model oversight committees and postdeployment monitoring programs.

a AI: artificial intelligence.

Economic feasibility represents an important consideration for broader adoption of governance-oriented implementation models [43]. The framework does not require the independent construction of large-scale computational infrastructure. The operative condition is continuity of access to governed data and computational resources rather than infrastructural ownership. In practice, this requirement may be fulfilled through shared regional platforms, cloud-based infrastructures operating under institutional data governance agreements, or staged implementation strategies focusing on high-value clinical pathways. These configurations allow institutions across resource levels to establish governance capacity without replicating the infrastructural scale characteristic of tertiary referral centers. Resource pooling, coordinated implementation, and shared accountability structures may therefore support deployment across diverse institutional configurations, including regional health networks, public-private partnerships, and externally supported platforms.

This functional interpretation situates the framework within the broader landscape of international AI governance initiatives while clarifying its distinct contribution. Existing frameworks, including ISO/IEC 42001 [44], World Health Organization guidance on health AI governance [19], and regulatory pathways developed by agencies such as the US Food and Drug Administration [45], share a common structural assumption that the organizational capacity to implement governance already exists within deploying institutions. These frameworks specify what governance should achieve, including accountability documentation, risk management structures, and compliance requirements. However, they presuppose the existence of an institutional carrier capable of activating and sustaining these arrangements [25,28]. The framework developed here addresses the prior question of how such capacity comes into existence. It identifies the conditions under which organizational ownership, coordination authority, and oversight mechanisms emerge through implementation rather than being fully established in advance [22,24]. These two levels should therefore be seen as complementary rather than competing. Normative standards define the accountability architecture within which clinical AI should operate, while the present framework explains the institutional formation process through which that architecture becomes operationally viable at the point of deployment. The framework is therefore transferable not because institutions can or should replicate the Hebei case in structural detail, but because comparable governance functions may be identified, examined, and adapted across heterogeneous implementation settings.

### From Viewpoint to Research Agenda

The framework presented in this study derives from a single institutional case observed during an early stage of governance consolidation, which necessarily constrains generalizability [46]. Embedded observation enabled detailed insight into implementation dynamics but also introduced interpretive proximity. Triangulation across traceable institutional artifacts and iterative review by coauthors with distinct clinical, administrative, and governance roles were used to mitigate this limitation. Lifecycle governance mechanisms were still evolving during the observation period, and participatory governance remained only partially developed. The scope of claims the present analysis can support is accordingly bounded by these conditions. Although the framework may be analytically transferable across settings, its empirical adequacy and practical consequences remain to be tested through comparative research.

Beyond the constraints of this single case, a more fundamental limitation lies in the current state of the field. Clinical AI governance research has generated a substantial body of normative frameworks, implementation principles, and policy guidance, yet the relationship between governance arrangements and clinical outcomes remains largely unexamined [47]. Governance frameworks are frequently justified by the assumption that they improve care quality, patient safety, and operational reliability. However, empirical investigation of this assumption remains conspicuously limited. Governance studies rarely measure clinical outcomes, and outcome studies rarely examine governance structures [47]. The result is a structural circularity in which the value of governance is asserted but seldom demonstrated empirically.

Breaking this circularity requires a specific methodological intervention, namely longitudinal comparative studies that track governance formation and clinical outcomes simultaneously, treating governance artifacts, including carrier establishment, authorization records, coordination protocols, and oversight documentation, as traceable intermediate variables in a causal pathway from implementation to clinical impact [48]. Investigations of this kind would need to span multiple institutional contexts to distinguish governance effects from institutional confounders and would require follow-up periods sufficient to capture the maturation of lifecycle governance mechanisms beyond early implementation phases. Economic analyses characterizing the cost structures of alternative governance configurations constitute an equally pressing methodological demand [49], particularly for institutions operating with resource constraints that preclude replication of tertiary-center infrastructure. Most fundamentally, the field requires an agreed methodology for attributing clinical and operational outcomes to governance arrangements rather than to technical model performance alone [14,47], a distinction that existing evaluation frameworks are currently ill-equipped to draw.

### Conclusions

Clinical AI adoption is typically framed as a problem of technology transfer, understood as the movement of capable systems from development into clinical practice. The present analysis points toward a more fundamental reconceptualization. What health systems are required to construct is not a pathway for technology to enter institutions but the institutional capacity through which technology becomes governable, accountable, and sustained. Institutional capacity of this kind is not established in advance of implementation; it is generated through it. The operational tensions that arise during deployment are not obstacles to governance formation. They are its generative conditions. The 6-module framework developed in this study identifies the functional arrangements through which this formation process stabilizes into sustained institutional capacity. The framework thus reorients institutional strategy away from governance prespecification and toward the cultivation of implementation conditions capable of generating governance over time. Institutions that recognize governance as a product of implementation rather than its prerequisite are better positioned to support the iterative process through which clinical AI transitions from experimental artifact to organizational infrastructure. Without such capacity, technically mature systems remain perpetually promising pilots awaiting conditions that never arrive.

## Abbreviations

AI: artificial intelligence
EMR: electronic medical record
MDT: multidisciplinary team
TDM: therapeutic drug monitoring
